# Long-term Mortality Trends Among Individuals With Tuberculosis: A Retrospective Cohort Study of Individuals Diagnosed With Tuberculosis in Brazil

**DOI:** 10.1093/cid/ciaf206

**Published:** 2025-04-18

**Authors:** Sun Kim, Daniele M Pelissari, Luiza O Harada, Mauro Sanchez, Patricia Bartholomay Oliveira, Fernanda D C Johansen, Ethel L N Maciel, Ted Cohen, Marcia C Castro, Nicolas A Menzies

**Affiliations:** Department of Global Health and Population, Harvard T. H. Chan School of Public Health, Boston, Massachusetts, USA; Health and Environment Surveillance Secretariat, Ministry of Health, Brasília, Brazil; Health and Environment Surveillance Secretariat, Ministry of Health, Brasília, Brazil; Department of Public Health, University of Brasília, Brasília, Brazil; Health and Environment Surveillance Secretariat, Ministry of Health, Brasília, Brazil; Health and Environment Surveillance Secretariat, Ministry of Health, Brasília, Brazil; Health and Environment Surveillance Secretariat, Ministry of Health, Brasília, Brazil; Department of Epidemiology of Microbial Diseases, Yale School of Public Health, New Haven, Connecticut, USA; Department of Global Health and Population, Harvard T. H. Chan School of Public Health, Boston, Massachusetts, USA; Department of Global Health and Population, Harvard T. H. Chan School of Public Health, Boston, Massachusetts, USA

**Keywords:** posttuberculosis, long-term mortality, tuberculosis survivors, clinical factors, cause of death

## Abstract

**Background:**

Even after successful treatment, individuals surviving tuberculosis (TB) disease experience elevated mortality rates. However, there is limited evidence on how these risks vary over time and by individual characteristics.

**Methods:**

We conducted a retrospective cohort study of individuals diagnosed with TB in Brazil, using national TB notifications and linked mortality records for 2007–2016. We estimated mortality rate ratios (MRRs) and cumulative mortality by year since TB diagnosis, compared to general population mortality matched on age, sex, year, and state. We identified clinical and sociodemographic factors associated with elevated post-TB mortality, and compared the distribution of causes of death to the general population.

**Results:**

The study sample included 834 594 individuals, with 4.1 million person-years of follow-up (average, 4.9 years). The TB cohort had elevated mortality compared to the general population, particularly in the first year postdiagnosis (MRR, 11.28 [95% confidence interval {CI}, 11.18–11.37]). Post-TB MRRs declined from 3.59 (95% CI, 3.53–3.64) in year 2 to 1.46 (95% CI, 1.34–1.59) in year 10. Cumulative excess mortality was 6.12% (95% CI, 6.07%–6.17%) after 1 year and 9.90% (95% CI, 9.58%–10.24%) after 10 years. MRRs were highest for individuals 30–44 years old at diagnosis. Relapse, loss to follow-up, and co-prevalent conditions like human immunodeficiency virus (HIV) and alcohol use disorder were strongly associated with higher MRRs. Over time, major causes of death in the TB cohort shifted from TB and HIV to cardiovascular disease, cancer, and non-TB respiratory diseases.

**Conclusions:**

Individuals developing TB disease face elevated mortality up to 10 years after diagnosis. These excess risks vary across demographic and clinical characteristics.

## Teaser

A retrospective cohort study of 834 594 individuals with tuberculosis in Brazil (2007–2016) revealed elevated mortality up to 10 years postdiagnosis, particularly in the first year. Mortality risks varied by age and co-prevalent conditions, and shifted from TB to chronic diseases.

Tuberculosis (TB) was responsible for 1.25 million deaths in 2023 [1]. Although effective treatment reduces TB-related deaths, observational studies have consistently estimated higher all-cause mortality rates for TB survivors compared to the general population [2–6]. This elevated post-TB mortality results from chronic sequelae caused by TB [7–9], as well as preexisting differences in health status, behaviors, and social conditions between individuals who develop TB and the general population [10]. However, there is limited evidence describing how the excess mortality faced by TB survivors varies over time since TB or by individual characteristics. Given the substantial number of individuals surviving TB disease [11] and the significant health losses in this population [12], better evidence on long-term mortality trends among TB survivors could allow better quantification of future health risks and identify clinical and patient factors that could be targeted to improve outcomes.

In this study, we estimated long-term mortality trends among individuals diagnosed with TB in Brazil between 2007 and 2016. For this TB cohort we estimated differences in mortality rates over time since TB diagnosis as compared to the general population, and analyzed how mortality trends differed by age group, sex, and other sociodemographic and clinical factors. We calculated differences in cumulative mortality to assess how deaths during the TB episode and post-TB period contributed to overall excess mortality and describe how causes of death changed over time.

## METHODS

### Data

We utilized a linked dataset of TB case notifications from Brazil's National Notifiable Disease Information System (Sistema de Informação de Agravos de Notificação [SINAN]) [13] for 2007–2016, and death records for individuals included this dataset, as recorded in the Brazilian Mortality Information System (Sistema de Informação sobre Mortalidade [SIM]) [14], covering the same period. Data linkage was conducted by Brazilian Ministry of Health personnel [15]. From this linked dataset we extracted diagnosis date, age at diagnosis, sex, self-reported race, state of residence, educational attainment, co-prevalent medical and social conditions (human immunodeficiency virus [HIV], alcohol use disorder, diabetes, mental illness, incarceration), and clinical variables (diagnostic test results, type of TB [pulmonary/extrapulmonary], chest X-ray result, recorded treatment outcome [cured, loss to follow-up, transfer, treatment failure, drug resistance, other]). For deaths during follow-up we recorded the date and cause of death.

We excluded individuals whose TB diagnosis was subsequently revised to a different disease diagnosis (assumed to not have TB) and individuals diagnosed with TB postmortem. Additionally, we excluded records with missing age, sex, or state, and individuals aged ≥90 years.

Individuals were censored at the point of death and otherwise assumed to remain in the study cohort. This approach assumed that (1) individuals did not emigrate (since deaths occurring outside Brazil would not appear in SIM), and (2) deaths were recorded in SIM with sufficient detail to be linked to a SINAN record. The Institutional Review Board of the Harvard T. H. Chan School of Public Health determined that this study is not human subjects research (protocol number IRB23-0844).

For the general population, we extracted reported deaths from SIM for 2007–2016 along with population data for the corresponding year, age, sex, and state from the Brazilian Institution of Geography and Statistics (Instituto Brasileiro de Geografia e Estatística) [16]. Using these data, we fitted generalized additive regression models to create smoothed mortality estimates for the general population stratified by year, age, sex, and state.

### Outcomes of Interest

The primary outcome was mortality rate ratios (MRRs) for the TB cohort compared to the general population, by year since TB diagnosis. We estimated MRRs for the overall cohort, as well as by age group, sex, and region. We defined the “TB episode” as the 12 months following TB diagnosis, and the “post-TB period” as any observation time >12 months following diagnosis [6]. We fit univariable and multivariable regression models for mortality during the TB episode and during the post-TB period, respectively, to estimate the relationship between MRRs and a range of sociodemographic and clinical factors, including sex, bacteriological test results, type of TB (pulmonary vs extrapulmonary), chest X-ray, type of entry (new case or relapse), comorbid conditions, diagnosis in prison, and recorded treatment outcome (cured, loss to follow-up, treatment failure, transfer, change in treatment, and unknown).

Using the estimated MRRs and general population mortality, we estimated cumulative mortality over time for the TB cohort compared to the general population and compared these curves to quantify the excess mortality experienced by the TB cohort. To quantify the contribution of deaths during the TB episode and the post-TB period to overall excess mortality, we estimated excess mortality under an additional counterfactual scenario that only applied TB-associated MRRs over the TB episode.

We categorized deaths into 11 groups (TB, HIV, respiratory disease, infectious disease, trauma, endocrine/metabolic disorder, digestive disease, cardiovascular disease, cancer, other, and unknown) based on *International Classification of Diseases, Tenth Revision* code. For the TB cohort, the proportion of deaths by cause was calculated for each year since TB diagnosis. We compared these results to the distribution of deaths in the general population matched by age, sex, state, and calendar year.

### Statistical Analysis

Study outcomes were estimated from generalized linear regression models fit with a complementary log-log link function and an offset term calculated as the log of the expected number of deaths in the general population. In this regression framework, the exponentiated regression coefficients represent MRRs for the TB cohort compared to the general population. We estimated 95% confidence intervals (CIs) by simulating from the uncertainty distributions of the model coefficients. Analyses were conducted in R version 4.4.1 software [17], using the mgcv package v1.9-1 [18].

## RESULTS

Over 2007–2016 there were 861 928 recorded TB cases. After excluding cases with unknown age (n = 344), age ≥90 years (n = 1725), missing sex (n = 53), missing state of residence (n = 324), postmortem diagnosis (n = 6041), or a recorded change in diagnosis (n = 18 847), the final dataset included 834 594 individuals with TB. The average duration of follow-up was 4.9 years, representing 4 089 883 person-years of follow-up. There were 120 330 deaths during follow-up (14% of individuals in the cohort). We reported our results following the REporting of studies Conducted using Observational Routinely-collected Data (RECORD) statement (Supplementary Table 1) [19]. Table 1 summarizes the study cohort, Supplementary Table 2 provides additional descriptive statistics for covariates not listed in Table 1, and Supplementary Table 3 reports the prevalence of comorbid conditions by age group.

**Table 1.** Characteristics of the Cohort

Characteristic	Individuals in Cohort, No.	All-Cause Deaths at the End of Follow-up, No. (%)	Person-years of Follow-up
Total	834 594	120 330 (14.4)	4 089 883
Year of diagnosis			
2007–2010	263 363	61 033 (23.2)	2 426 476
2011–2013	221 066	36 919 (16.7)	1 180 657
2014–2016	229 835	22 378 (9.7)	482 750
Age group, y			
0–14	25 077	1014 (4.0)	143 924
15–29	228 803	15 187 (6.6)	1 275 255
30–44	229 154	34 537 (15.1)	1 305 020
45–59	156 140	36 782 (23.6)	913 634
60–74	60 381	22 897 (37.9)	357 680
75–89	14 709	9913 (67.4)	94 370
Sex			
Female	240 417	30 295 (12.6)	1 377 953
Male	473 847	90 035 (19.0)	2 711 930
Race			
White	230 953	40 539 (17.6)	1 339 033
Black	92 495	16 843 (18.2)	531 097
Asian	6036	927 (15.3)	37 289
Brown	306 095	47 720 (15.6)	1 668 724
Indigenous	8271	772 (9.3)	46 454
Unknown	70 414	13 529 (19.2)	467 286

### Mortality Rate Ratios

**Figure 1.**
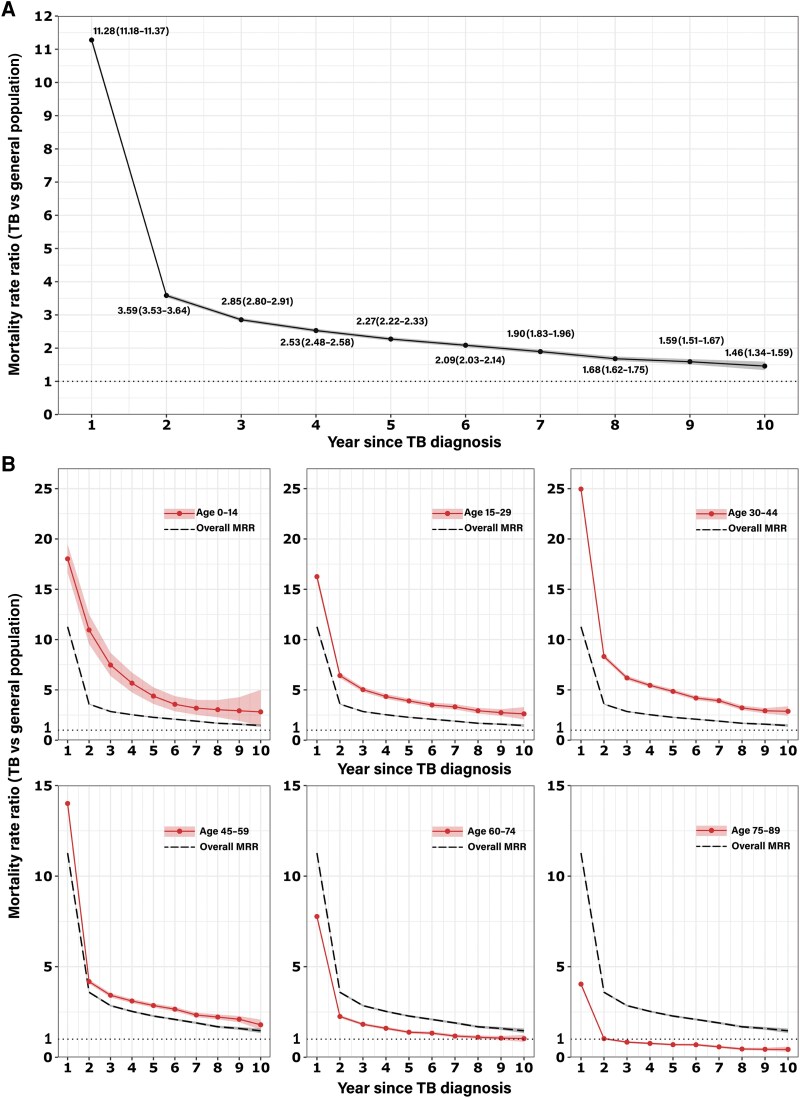
Mortality rate ratios (MRRs) for individuals with tuberculosis (TB) by year since TB diagnosis, for the overall TB cohort (*A*) and by age group at TB diagnosis (*B*), as compared to the general population. In *B*, the solid lines indicate the MRR for each age group, and the dashed lines show MRRs estimated for the overall population for reference. Dotted line represents MRR = 1.0 (no excess mortality).

Mortality rates in the TB cohort varied by age group and years since diagnosis. Figure 1*A* reports MRRs for individuals in the TB cohort compared to the general population, up to 10 years following diagnosis. During the TB episode (first year following diagnosis), the MRR in the overall cohort was estimated as 11.28 (95% CI, 11.18–11.37). This dropped to 3.59 (95% CI, 3.53–3.64) in the second year and continued to decline monotonically, reaching 2.27 (95% CI, 2.22–2.33) in the fifth year and 1.46 (95% CI, 1.34–1.59) in the tenth year after diagnosis. Figure 1*B* presents MRRs by age group. For the first year since diagnosis, MRRs ranged between 4.04 (95% CI, 3.94–4.15) for individuals 75–89 years old at diagnosis and 24.97 (95% CI, 24.59–25.33) for individuals 30–44 years old at diagnosis. For each age group, MRRs declined rapidly from the first to the second year following diagnosis, then more slowly over subsequent years. In general, MRRs were highest for 30- to 44-year-olds, and lowest for 75- to 89-year-olds. Among the age group 75–89 years, MRRs were <1.0 (indicating lower mortality) after the second year following diagnosis (Supplementary Table 4).

Supplementary Figure 1 presents MRRs by sex. In the first year following diagnosis, the MRR was higher for females (13.59 [95% CI, 13.37–13.80]) compared to males (10.61 [95% CI, 10.51–10.72]). However, this trend reversed by the third year, with males showing higher MRRs, a pattern that continued through year 10. MRRs by year and sex are reported in Supplementary Table 5. Supplementary Figure 2 presents MRRs by region. The South region had the highest overall MRR (15.28 [95% CI, 14.96–15.60] in the first year), while the North region showed the lowest MRR (9.79 [95% CI, 9.51–10.06] in the first year). MRRs by year and region are detailed in Supplementary Table 6.

### Predictors of TB Episode and Post-TB Mortality

The factor most strongly associated with increased mortality during the TB episode was a positive HIV diagnosis (adjusted MRR [aMRR], 5.16 [95% CI, 5.05–5.27], compared to HIV negative). Female sex (aMRR, 1.67 [95% CI, 1.64–1.70], compared to male sex), combined pulmonary and extrapulmonary TB (aMRR, 1.57 [95% CI, 1.52–1.62], compared to pulmonary TB), and X-ray evidence of other lung pathology (aMRR, 1.77 [95% CI, 1.65–1.89], compared to normal X-ray) were also associated with elevated mortality (Supplementary Table 7).

Factors associated with elevated post-TB mortality included female sex (aMRR, 1.41 [95% CI, 1.38–1.43], compared to male sex), reinitiation of treatment following abandonment (aMRR, 1.78 [95% CI, 1.74–1.84], compared to new cases), positive HIV diagnosis (aMRR, 2.80 [95% CI, 2.73–2.87], compared to HIV negative), and a history of alcohol use disorder (aMRR, 1.52 [95% CI, 1.49–1.55], compared to those without such history), as well as loss to follow-up and cases with a change in regimen due to drug resistance (aMRR, 1.68 [95% CI, 1.64–1.72] and 1.65 [95% CI, 1.53–1.77], respectively, compared with treatment outcome of cure) (Table 2).

**Table 2.** Predictors of Posttuberculosis Mortality

Predictor	Univariable Analysis	Multivariable Analysis	
	MRR (95% CI)	aMRR (95% CI)	Without Treatment Outcome, aMRR (95% CI)
Sex			
Male	Reference	Reference	Reference
Female	1.22 (1.20–1.25)	1.41 (1.38–1.43)	1.39 (1.37–1.42)
Bacteriological test results			
Negative	Reference	Reference	Reference
Positive (smear or culture or Xpert)	0.99 (.97–1.01)	.99 (.97–1.01)	0.99 (.97–1.02)
Not performed	0.93 (.90–.95)	0.97 (.94–.99)	0.98 (.95–1.01)
Type of TB			
Pulmonary	Reference	Reference	Reference
Extrapulmonary	0.83 (.81–.85)	0.89 (.86–.92)	0.87 (.85–.90)
Pulmonary + extrapulmonary	1.34 (1.29–1.40)	1.04 (1.00–1.09)	1.04 (1.00–1.09)
Unknown	0.91 (.53–1.57)	1.25 (.72–2.17)	1.16 (.67–2.00)
Chest X-ray			
Normal	Reference	Reference	Reference
Suspected	1.20 (1.15–1.25)	1.19 (1.14–1.24)	1.19 (1.14–1.25)
Other pathology	1.19 (1.07–1.31)	1.20 (1.08–1.33)	1.21 (1.09–1.34)
Not performed	1.13 (1.08–1.18)	1.11 (1.06–1.17)	1.12 (1.07–1.17)
Type of entry			
New case	Reference	Reference	Reference
Relapse	1.73 (1.68–1.77)	1.52 (1.48–1.56)	1.58 (1.54–1.62)
Reentry after abandonment	2.51 (2.44–2.57)	1.78 (1.74–1.84)	2.08 (2.03–2.14)
Unknown	1.10 (.93–1.30)	1.00 (.84–1.19)	1.05 (.89–1.25)
Transfer	1.32 (1.27–1.37)	1.18 (1.14–1.23)	1.23 (1.18–1.28)
Comorbid conditions			
HIV	2.98 (2.91–3.04)	2.80 (2.73–2.87)	2.94 (2.87–3.02)
Alcohol use disorder	1.64 (1.60–1.67)	1.52 (1.49–1.55)	1.58 (1.54–1.61)
Diabetes	1.25 (1.22–1.29)	1.37 (1.33–1.41)	1.36 (1.32–1.40)
Mental illness	1.39 (1.32–1.45)	1.20 (1.14–1.26)	1.20 (1.14–1.26)
In prison (at diagnosis)			
No	Reference	Reference	Reference
Yes	1.04 (1.00–1.07)	1.08 (1.04–1.12)	1.05 (1.01–1.09)
Unknown	0.92 (.88–.96)	0.98 (.94–1.03)	0.98 (.94–1.02)
Recorded treatment outcome			
Cured	Reference	Reference	
Loss to follow-up	2.08 (2.04–2.12)	1.68 (1.64–1.72)	
Treatment failure	.79 (.29–2.10)	.73 (.27–1.94)	
Transfer	1.47 (1.43–1.52)	1.32 (1.28–1.36)	
Drug resistance + change in treatment (from fixed dose combination)	2.19 (2.04–2.35)	1.65 (1.53–1.77)	
Unknown	1.10 (1.04–1.16)	1.06 (1.00–1.12)	

Post-TB period is defined as >12 months after TB diagnosis. All results are adjusted for the interaction between age group and years since TB diagnosis, along with race, state, and educational attainment.

Abbreviations: aMRR, adjusted mortality rate ratio; CI, confidence interval; HIV, human immunodeficiency virus; MRR, mortality rate ratio; TB, tuberculosis.

### Cumulative Mortality

**Figure 2.**
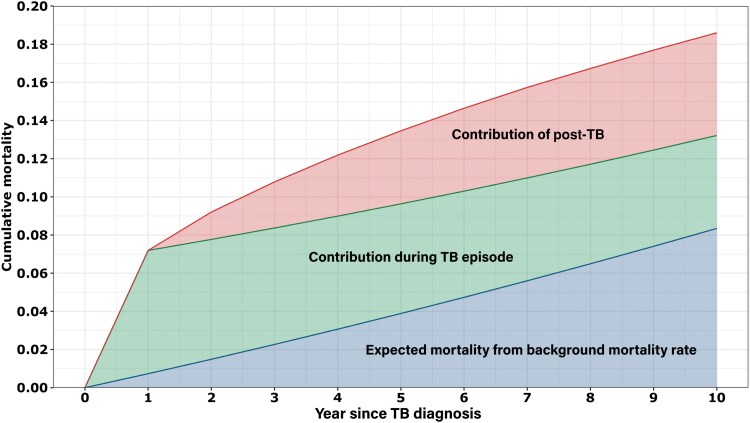
Cumulative mortality by year since tuberculosis (TB) diagnosis. The bottom area shows the baseline mortality rate expected in the general population without TB, the middle area represents the contribution of deaths during the TB episode to overall excess mortality, and the top area indicates the contribution of mortality during the post-TB period to overall excess mortality.

Figure 2 compares cumulative mortality for the TB cohort and the general population over the follow-up period. In the first year, individuals in the TB cohort had a cumulative mortality of 6.82% (95% CI, 6.77%–6.86%), compared to 0.70% for the general population. Excess mortality for the TB cohort was 6.12% (95% CI, 6.07%–6.17%) at the end of the first year following diagnosis. Excess mortality increased over the post-TB period: By year 5, cumulative mortality for the TB cohort reached 12.87% (95% CI, 12.73%–13.01%), compared to 3.69% in the general population, with 9.18% (95% CI, 9.04%–9.32%) excess mortality. By the end of year 10, cumulative mortality for the TB cohort was estimated as 17.88% (95% CI, 17.55%–18.21%), compared to 7.97% among the general population. At this point total excess mortality was 9.90% (95% CI, 9.58%–10.24%), with 5.22% (95% CI, 4.90%–5.56%) of this total resulting from post-TB mortality. Across age groups, those 45–59 years had the greatest excess mortality after 10 years (18.63%) (Supplementary Table 8 and Supplementary Figure 3). Males were estimated to have consistently higher excess mortality than females, with 11.32% (95% CI, 10.90%–11.76%) excess mortality after 10 years, versus 6.95% (95% CI, 6.52%–7.40%) for females (Supplementary Table 9 and Supplementary Figure 4). Across regions, the South showed the highest excess mortality, reaching 13.84% (95% CI, 12.89%–14.83%) after 10 years (Supplementary Table 10 and Supplementary Figure 5).

### Cause of Death

**Figure 3.**
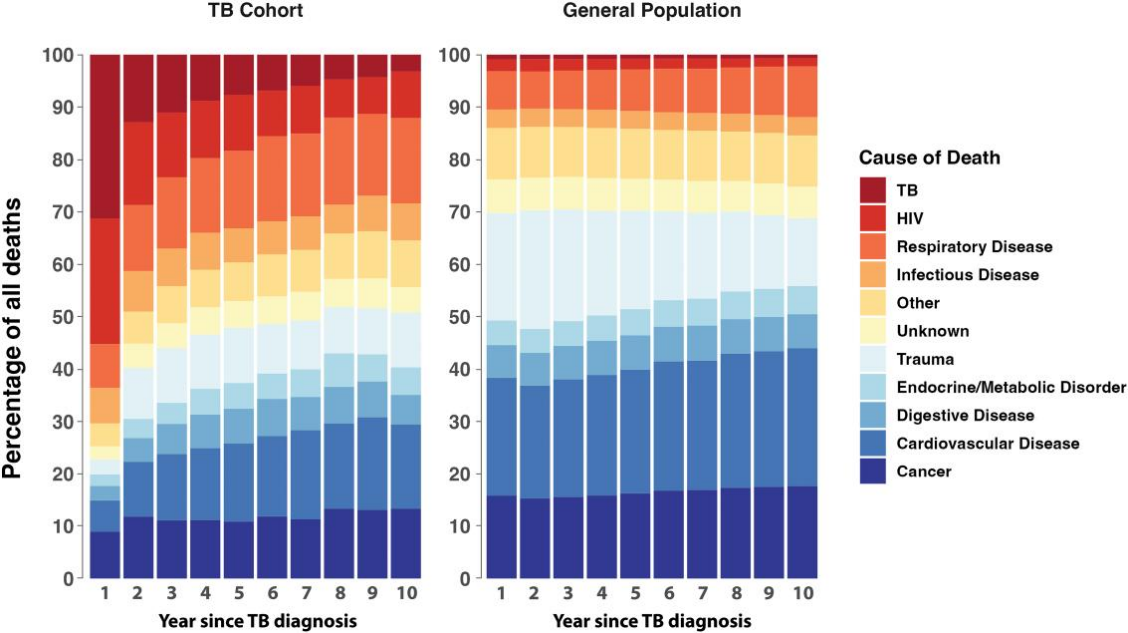
Distribution of cause of death in the tuberculosis (TB) cohort compared to a matched general population sample, by years since TB diagnosis. Distribution of deaths in the general population created by weighting deaths in the Brazilian Mortality Information System to match the distribution of age, sex, state of residence, and calendar year for deaths in the TB sample, for each year since diagnosis. Abbreviations: HIV, human immunodeficiency virus; TB, tuberculosis.

Causes of death differed markedly between the TB cohort and a matched general population sample. Figure 3 compares causes of death between these 2 populations, by year since TB diagnosis. In the first year after diagnosis, TB and HIV were the leading causes of death in the TB cohort, accounting for 31.3% and 23.9% of total deaths, respectively, compared to 0.9% and 2.3% in the general population. In the tenth year after diagnosis, respiratory diseases (16.3%), cardiovascular diseases (16.1%), and cancer (13.3%) were the top causes of death for the TB cohort, compared to 9.6% for respiratory diseases, 26.3% for cardiovascular diseases, and 17.7% for cancer in the general population. Supplementary Tables 11 and 12 show cardiovascular and respiratory disease deaths further stratified into finer categories, and Supplementary Figure 6 shows causes of death by region.

## DISCUSSION

In this study we assessed mortality rates among a large cohort of individuals with TB disease, up to 10 years after initial diagnosis. We found these individuals to have significantly higher mortality than the general population, with mortality >11 times higher in the first year after diagnosis and remaining elevated for up to 10 years after diagnosis. This extended period of elevated mortality highlights the long-term health challenges faced by TB survivors, reinforcing findings from previous studies [5, 11, 12]. A meta-analysis by Romanowski et al reported a pooled standardized mortality ratio of 2.91 for all-cause mortality in TB cohorts compared to the general population [5]. While our results show similar increases in mortality for the cohort overall, they also reveal substantial variations over the post-TB period by patient characteristics.

MRRs estimated for each age group followed similar time trends, although with notable differences in magnitude, with the age group 30–44 years exhibiting MRRs >2 times greater than the overall average. This may in part be due to the elevated prevalence of HIV in this age group (17.9% vs 8.4% in other age groups), which is a strong predictor of TB-related mortality. In contrast, the age group 75–89 had the lowest MRRs, potentially related to elevated baseline mortality, as well as survivorship bias [20].

This study also identified key predictors of long-term mortality among individuals who survived the first 12 months after TB diagnosis (post-TB). Relapse, loss to follow-up, and drug-resistant TB were each strongly associated with increased mortality within the TB cohort, reflecting the harmful effects of interrupted or unsuccessful TB treatment [21–23]. In addition, HIV infection was one of the strongest predictors of mortality, with HIV-positive individuals having a post-TB mortality 2.83 times that of HIV-negative individuals. This finding reinforces the well-established interaction between TB and HIV, with HIV increasing both the risk of developing TB and the risk of TB progression and mortality among individuals with active TB disease [24–26]. In addition to HIV, individuals with a history of alcohol use disorder had a higher risk of death. This could be due to the harmful effects of alcohol on the immune system and its potential to worsen TB outcomes through poor treatment adherence and increased risk of complications [27, 28].

We also found MRRs to be higher for females compared to males. This result is surprising, given other study results finding females to have lower mortality during TB treatment [29, 30], and the difference remained after controlling for a range of other patient covariates. Part of the explanation for this finding may relate to differences in background mortality, with males in the general population experiencing substantially higher mortality compared to females. Due to these differences, excess mortality estimates for males were 63% higher than those for females, despite the higher MRR estimated for females. This result could also indicate differences in the incidence and severity of post-TB sequelae between men and women, and further research on this question would be valuable.

The cumulative mortality estimates highlight both the immediate and long-term excess mortality among TB survivors. While there was substantial excess mortality in the TB cohort during the first 12 months following diagnosis, mortality during the post-TB period represented 53% of overall excess mortality. This finding aligns with recent global estimates of the lifetime burden of TB disease, which attributed 47% of the total TB-related disease burden in 2019 to post-TB sequelae [12].

Last, the cause of death analysis highlights the evolving health risks for TB survivors. In the first year after diagnosis, TB and HIV were leading causes of death. By the tenth year, however, the main causes of death among TB survivors had shifted to non-TB respiratory diseases, cardiovascular diseases, and cancer. Although the proportion of deaths due to cardiovascular diseases is lower in the TB cohort compared to the general population, this is explained by the elevated all-cause mortality in the TB cohort. This is consistent with recent findings indicating that a substantial portion (20%) of posttreatment deaths are attributable to cardiovascular disease [5]. This transition in causes of death among the TB cohort—from TB and HIV in the early years to chronic conditions in later years—reflects the complex health challenges faced by TB survivors. These findings reflects not only the immediate risks posed by TB but also the long-term consequences of TB sequelae, which increase survivors' susceptibility to other diseases, including noncommunicable diseases [31]. Over the post-TB period, a high proportion of deaths were attributed to non-TB respiratory causes, suggesting elevated susceptibility to or severity of these conditions among TB survivors. This underscores the importance of ongoing healthcare support and preventive strategies after the completion of TB treatment, tailored to the pattern of sequelae and health risks faced by TB survivors [32].

This study has several limitations. First, our results rely on the accuracy of SINAN and SIM data. Any misclassification of TB status or cause of death in these data could bias our estimates. Second, while we defined the “post-TB period” as beginning 12 months following diagnosis, individuals with drug-resistant TB may receive regimens of 18–24 months in length. We chose 12 months to have a consistent definition for all patients, and because patients receiving long regimens represent a small minority of the treatment cohort. Third, we were unable to account for individuals who may have emigrated from Brazil during the study period. However, emigration rates for Brazil are low (0.8% in 2019) [33], and it is therefore unlikely that emigration had a major impact on results. Fourth, while we were able to distinguish patients according to whether or not they were diagnosed with drug-resistant TB, we were not able to further stratify results by the pattern of drug resistance for each patient. Similarly, while we estimated differences in mortality by the presence/absence of lung pathology at TB diagnosis, we were not able to assess relative impact of more or less extensive lung involvement. Finally, while our study adjusted for age, sex, state, and year of death to calculate the MRRs of the TB cohort compared to the general population's background mortality, we were unable to match on other socioeconomic or demographic variables, such as income or occupation. These unmeasured factors may have contributed to the mortality differences observed between the groups, indicating that our results reflect not only the causal effect of TB, but also the combined effect of TB and these underlying socioeconomic determinants.

Despite these limitations, this study represents one of the most comprehensive analyses of long-term survival following TB, and it is among the first to explore how MRRs of TB survivors vary by years since diagnosis compared to the general population. Drawing on data from >800 000 individuals and >4 million years of follow-up, we were able to examine excess mortality across a wide range of patient characteristics—including age, sex, and clinical and demographic factors—at a level of detail not possible in smaller studies. The findings from our study highlight the ongoing healthcare needs of TB survivors, a challenge not unique to Brazil but relevant to many other countries facing significant TB burdens [10].

## Supplementary Material

ciaf206_Supplementary_Data

## Supplementary Data

Supplementary materials are available at *Clinical Infectious Diseases* online. Consisting of data provided by the authors to benefit the reader, the posted materials are not copyedited and are the sole responsibility of the authors, so questions or comments should be addressed to the corresponding author.

## Notes

***Data availability.*** Secondary and de-identified data from SIM and SINAN are available online on the official webpage of the Ministry of Health of Brazil. These data can be accessed at: https://datasus.saude.gov.br/transferencia-de-arquivos/.

***Financial support***. This work was supported by the National Institute of Allergy and Infectious Diseases of the National Institutes of Health (award number R01AI146555 to N. A. M.).
