# Supplementary material for: Long-term Mortality Trends Among Individuals With Tuberculosis: A Retrospective Cohort Study of Individuals Diagnosed With Tuberculosis in Brazil
Source: Clin Infect Dis. 2025 Apr 18;82(1):e1–8. doi: 10.1093/cid/ciaf206 (PMC13375577; doi:10.1093/cid/ciaf206)
Supplement: ciaf206_Supplementary_Data [file ciaf206_supplementary_data.docx]

**SUPPLEMENT**

**Table S1. The RECORD statement – checklist of items, extended from the STROBE statement, that should be reported in observational studies using routinely collected health data.**

|  | **Item No.** | **STROBE items** | **Location in manuscript where items are reported** | **RECORD items** | **Location in manuscript where items are reported** |
| --- | --- | --- | --- | --- | --- |
| **Title and abstract** | | | | | |
|  | 1 | (a) Indicate the study’s design with a commonly used term in the title or the abstract (b) Provide in the abstract an informative and balanced summary of what was done and what was found |  | RECORD 1.1: The type of data used should be specified in the title or abstract. When possible, the name of the databases used should be included.  RECORD 1.2: If applicable, the geographic region and timeframe within which the study took place should be reported in the title or abstract.  RECORD 1.3: If linkage between databases was conducted for the study, this should be clearly stated in the title or abstract. | Page 2 abstract  Page 2 abstract  Page 2 abstract |
| **Introduction** | | | | | |
| Background rationale | 2 | Explain the scientific background and rationale for the investigation being reported |  |  | Page 3 introduction |
| Objectives | 3 | State specific objectives, including any prespecified hypotheses |  |  | Page 3 introduction |
| **Methods** | | | | | |
| Study Design | 4 | Present key elements of study design early in the paper |  |  | Pages 5–7 methods: data, outcomes of interest |
| Setting | 5 | Describe the setting, locations, and relevant dates, including periods of recruitment, exposure, follow-up, and data collection |  |  | Pages 5–7 methods: data, outcomes of interest |
| Participants | 6 | *(a) Cohort study* - Give the eligibility criteria, and the sources and methods of selection of participants. Describe methods of follow-up  *Case-control study* - Give the eligibility criteria, and the sources and methods of case ascertainment and control selection. Give the rationale for the choice of cases and controls  *Cross-sectional study* - Give the eligibility criteria, and the sources and methods of selection of participants  *(b) Cohort study* - For matched studies, give matching criteria and number of exposed and unexposed  *Case-control study* - For matched studies, give matching criteria and the number of controls per case |  | RECORD 6.1: The methods of study population selection (such as codes or algorithms used to identify subjects) should be listed in detail. If this is not possible, an explanation should be provided.  RECORD 6.2: Any validation studies of the codes or algorithms used to select the population should be referenced. If validation was conducted for this study and not published elsewhere, detailed methods and results should be provided.  RECORD 6.3: If the study involved linkage of databases, consider use of a flow diagram or other graphical display to demonstrate the data linkage process, including the number of individuals with linked data at each stage. | Pages 5–7 methods: data, outcomes of interest, statistical analysis  Pages 5–11 methods & results, Table 1 & Table S2  Page 5 methods & citation #15 |
| Variables | 7 | Clearly define all outcomes, exposures, predictors, potential confounders, and effect modifiers. Give diagnostic criteria, if applicable. |  | RECORD 7.1: A complete list of codes and algorithms used to classify exposures, outcomes, confounders, and effect modifiers should be provided. If these cannot be reported, an explanation should be provided. | Pages 5–7 methods: data, outcomes of interest |
| Data sources/ measurement | 8 | For each variable of interest, give sources of data and details of methods of assessment (measurement).  Describe comparability of assessment methods if there is more than one group |  |  | Pages 5–7 methods: data, outcomes of interest |
| Bias | 9 | Describe any efforts to address potential sources of bias |  |  | Page 14 discussion |
| Study size | 10 | Explain how the study size was arrived at |  |  | Page 8 result first paragraph |
| Quantitative variables | 11 | Explain how quantitative variables were handled in the analyses. If applicable, describe which groupings were chosen, and why |  |  | Pages 5–7 methods: data, outcomes of interest |
| Statistical methods | 12 | (a) Describe all statistical methods, including those used to control for confounding  (b) Describe any methods used to examine subgroups and interactions  (c) Explain how missing data were addressed  (d) *Cohort study* - If applicable, explain how loss to follow-up was addressed  *Case-control study* - If applicable, explain how matching of cases and controls was addressed  *Cross-sectional study* - If applicable, describe analytical methods taking account of sampling strategy  (e) Describe any sensitivity analyses |  |  | Pages 5–7 methods: data, outcomes of interest, statistical analysis |
| Data access and cleaning methods |  | .. |  | RECORD 12.1: Authors should describe the extent to which the investigators had access to the database population used to create the study population.  RECORD 12.2: Authors should provide information on the data cleaning methods used in the study. | Pages 5–6 methods: data  Pages 5–6 methods: data |
| Linkage |  | .. |  | RECORD 12.3: State whether the study included person-level, institutional-level, or other data linkage across two or more databases. The methods of linkage and methods of linkage quality evaluation should be provided. | Page 5 methods & citation #15 |
| **Results** | | | | | |
| Participants | 13 | (a) Report the numbers of individuals at each stage of the study (*e.g.*, numbers potentially eligible, examined for eligibility, confirmed eligible, included in the study, completing follow-up, and analysed)  (b) Give reasons for non-participation at each stage.  (c) Consider use of a flow diagram |  | RECORD 13.1: Describe in detail the selection of the persons included in the study (*i.e.,* study population selection) including filtering based on data quality, data availability and linkage. The selection of included persons can be described in the text and/or by means of the study flow diagram. | Page 8 results, Table1 |
| Descriptive data | 14 | (a) Give characteristics of study participants (*e.g.*, demographic, clinical, social) and information on exposures and potential confounders  (b) Indicate the number of participants with missing data for each variable of interest  (c) *Cohort study* - summarise follow-up time (*e.g.*, average and total amount) |  |  | Page 8 results, Table1, Table S2 |
| Outcome data | 15 | *Cohort study* - Report numbers of outcome events or summary measures over time  *Case-control study* - Report numbers in each exposure category, or summary measures of exposure  *Cross-sectional study* - Report numbers of outcome events or summary measures |  |  | Page 8 results, Table1 |
| Main results | 16 | (a) Give unadjusted estimates and, if applicable, confounder-adjusted estimates and their precision (e.g., 95% confidence interval). Make clear which confounders were adjusted for and why they were included  (b) Report category boundaries when continuous variables were categorized  (c) If relevant, consider translating estimates of relative risk into absolute risk for a meaningful time period |  |  | Pages 9–11 results, Table 2, Figures 1–3 |
| Other analyses | 17 | Report other analyses done—e.g., analyses of subgroups and interactions, and sensitivity analyses |  |  | Tables S4–12, Figures S1–6 |
| **Discussion** | | | | | |
| Key results | 18 | Summarise key results with reference to study objectives |  |  | Pages 11–12 discussion |
| Limitations | 19 | Discuss limitations of the study, taking into account sources of potential bias or imprecision. Discuss both direction and magnitude of any potential bias |  | RECORD 19.1: Discuss the implications of using data that were not created or collected to answer the specific research question(s). Include discussion of misclassification bias, unmeasured confounding, missing data, and changing eligibility over time, as they pertain to the study being reported. | Page 14 discussion |
| Interpretation | 20 | Give a cautious overall interpretation of results considering objectives, limitations, multiplicity of analyses, results from similar studies, and other relevant evidence |  |  | Pages 11–13 discussion |
| Generalisability | 21 | Discuss the generalisability (external validity) of the study results |  |  | Page 15 discussion last paragraph |
| **Other Information** | | | | | |
| Funding | 22 | Give the source of funding and the role of the funders for the present study and, if applicable, for the original study on which the present article is based |  |  | Page 16 financial support |
| Accessibility of protocol, raw data, and programming code |  | .. |  | RECORD 22.1: Authors should provide information on how to access any supplemental information such as the study protocol, raw data, or programming code. | Page 16 notes: data availability |

*Reference: Benchimol EI, Smeeth L, Guttmann A, Harron K, Moher D, Petersen I, Sørensen HT, von Elm E, Langan SM, the RECORD Working Committee. The REporting of studies Conducted using Observational Routinely-collected health Data (RECORD) Statement. *PLoS Medicine* 2015; in press.

*Checklist is protected under Creative Commons Attribution ([CC BY](http://creativecommons.org/licenses/by/4.0/)) license.

**Table S2. Descriptive statistics of multivariable model covariates not listed in Table 1.**

| **Variable** | **Number of individuals**  **in cohort (N)** | **Percent of total cohort (%)** |
| --- | --- | --- |
| **Bacteriological test results** |  |  |
| Negative | 174,300 | 20.9 |
| Positive (smear or culture or Xpert) | 502,950 | 60.3 |
| Not performed | 157,344 | 18.9 |
| **Type of TB** |  |  |
| Pulmonary | 697,745 | 83.6 |
| Extrapulmonary | 109,142 | 13.1 |
| Pulmonary + Extrapulmonary | 27,421 | 3.3 |
| Unknown | 286 | 0.0 |
| **Chest Xray** |  |  |
| Normal | 42,979 | 5.1 |
| Suspected | 639,894 | 76.7 |
| Other pathology | 7,799 | 0.9 |
| Not performed | 143,922 | 17.2 |
| **Type of entry** |  |  |
| New case | 688,531 | 82.5 |
| Relapse | 56,806 | 6.8 |
| Reentry after abandonment | 56,939 | 6.8 |
| Unknown | 2,416 | 0.3 |
| Transfer | 29,902 | 3.6 |
| **Comorbid conditions** |  |  |
| HIV | 91,868 | 11.0 |
| Alcohol use disorder | 129,494 | 15.5 |
| Diabetes | 51,816 | 6.2 |
| Mental illness | 18,824 | 2.3 |
| **In prison (at diagnosis)** |  |  |
| No | 738,044 | 88.4 |
| Yes | 61,661 | 7.4 |
| Unknown | 34,889 | 4.2 |
| **Recorded treatment outcome**^*^ |  |  |
| Cured | 588,226 | 70.5 |
| Loss to follow-up | 105,708 | 12.7 |
| Treatment failure | 262 | 0.0 |
| Transfer | 522,263 | 6.3 |
| Drug resistance + change in  treatment (from fixed dose  combination) | 7,797 | 0.9 |
| Unknown | 20,357 | 2.4 |

^*^Treatment outcomes indicating death were excluded as mortality was the primary outcome

assessed in this study.

**Table S3. Prevalence of comorbid conditions in the TB cohort by age group**

|  | **HIV** | **Diabetes** | **Alcohol use disorder** | **Mental illness** |
| --- | --- | --- | --- | --- |
| **Overall (%)** | 11.00 | 6.21 | 15.52 | 2.26 |
| **Age group** |  |  |  |  |
| 0-14 | 4.58 | 0.95 | 1.72 | 1.22 |
| 15-29 | 8.06 | 1.07 | 8.38 | 1.64 |
| 30-44 | 17.93 | 3.70 | 19.94 | 2.55 |
| 45-59 | 10.67 | 11.53 | 22.72 | 2.89 |
| 60-74 | 3.42 | 16.51 | 13.20 | 2.10 |
| 75-89 | 1.17 | 13.05 | 4.78 | 1.75 |

**Table S4. Mortality rate ratios by year since diagnosis and age group**

|  | **Year 1**  **MRR (95% CI)** | **Year 2**  **MRR (95% CI)** | **Year 5**  **MRR (95% CI)** | **Year 10**  **MRR (95% CI)** |
| --- | --- | --- | --- | --- |
| **Overall** | 11.28 (11.18–11.37) | 3.58 (3.53–3.64) | 2.27 (2.22–2.33) | 1.46 (1.34–1.59) |
| **Age group** |  |  |  |  |
| 0-14 | 18.20 (16.84–19.65) | 10.36 (8.53–12.42) | 4.22 (3.00–5.76) | 1.95 (0.39–5.92) |
| 15-29 | 16.28 (15.90–16.66) | 6.37 (6.11–6.63) | 3.92 (3.67–4.18) | 2.63 (2.04–3.34) |
| 30-44 | 24.97 (24.59–25.33) | 8.29 (8.06–8.52) | 4.86 (4.65–5.07) | 2.87 (2.44–3.38) |
| 45-59 | 14.02 (13.81–14.22) | 4.16 (4.04–4.28) | 2.86 (2.74–2.97) | 1.78 (1.52–2.08) |
| 60-74 | 7.77 (7.64–7.91) | 2.24 (2.16–2.32) | 1.38 (1.31–1.46) | 1.03 (0.85–1.25) |
| 75-89 | 4.04 (3.94–4.15) | 1.02 (0.96–1.08) | 0.70 (0.63–0.75) | 0.43 (0.30–0.59) |

MRR = mortality rate ratio. CI = confidence interval.

**Table S5. Mortality rate ratios by year since diagnosis and sex**

|  | **Year 1**  **MRR (95% CI)** | **Year 2**  **MRR (95% CI)** | **Year 5**  **MRR (95% CI)** | **Year 10**  **MRR (95% CI)** |
| --- | --- | --- | --- | --- |
| **Overall** | 11.28 (11.18–11.37) | 3.58 (3.53–3.64) | 2.27 (2.22–2.33) | 1.46 (1.34–1.59) |
| **Sex** |  |  |  |  |
| Female | 13.59 (13.37–13.80) | 3.97 (3.85–4.10) | 2.15 (2.05–2.26) | 1.30 (1.09–1.55) |
| Male | 10.61 (10.51–10.72) | 3.48 (3.42–3.54) | 2.31 (2.25–2.37) | 1.52 (1.37–1.67) |

MRR = mortality rate ratio. CI = confidence interval.

**Table S6. Mortality rate ratios by year and region**

|  | **Year 1**  **MRR (95% CI)** | **Year 2**  **MRR (95% CI)** | **Year 5**  **MRR (95% CI)** | **Year 10**  **MRR (95% CI)** |
| --- | --- | --- | --- | --- |
| **Overall** | 11.28 (11.18–11.37) | 3.58 (3.53–3.64) | 2.27 (2.22–2.33) | 1.46 (1.34–1.59) |
| **Region** |  |  |  |  |
| North | 9.79 (9.51–10.06) | 3.00 (2.83–3.16) | 1.73 (1.59–1.89) | 1.10 (0.79–1.50) |
| Northeast | 9.88 (9.72–10.04) | 3.26 (3.16–3.36) | 2.02 (1.93–2.11) | 1.13 (0.93–1.35) |
| Central West | 10.77 (10.38–11.17) | 3.45 (3.22–3.70) | 2.24 (2.01–2.49) | 1.00 (0.60–1.59) |
| Southeast | 11.48 (11.34–11.62) | 3.56 (3.47–3.64) | 2.36 (2.28–2.44) | 1.74 (1.54–1.96) |
| South | 15.28 (14.96–15.60) | 5.01 (4.83–5.21) | 3.06 (2.88–3.25) | 1.82 (1.43–2.29) |

MRR = mortality rate ratio. CI = confidence interval.

**Table S7. Mortality rate ratios during the TB episode for patient characteristics.***

|  | Univariable –  (95% CI) | Multivariable – (95% CI) |
| --- | --- | --- |
| **Sex** |  |  |
| Male | Reference | Reference |
| Female | 1.59 (1.56–1.62) | 1.67 (1.64–1.70) |
| **Bacteriological test results** |  |  |
| Negative | Reference | Reference |
| Positive (smear or culture or Xpert) | 0.62 (0.61–0.63) | 0.72 (0.71–0.74) |
| Not performed | 1.21 (1.18–1.24) | 1.20 (1.18–1.23) |
| **Type of TB** |  |  |
| Pulmonary | Reference | Reference |
| Extrapulmonary | 1.43 (1.40–1.47) | 0.95 (0.93–0.98) |
| Pulmonary + Extrapulmonary | 2.83 (2.75–2.92) | 1.57 (1.52–1.62) |
| Unknown | 1.37 (0.94–2.00) | 1.11 (0.75–1.63) |
| **Chest Xray** |  |  |
| Normal | Reference | Reference |
| Suspected TB | 0.84 (0.81–0.87) | 1.18 (1.14–1.23) |
| Other pathology | 1.63 (1.52–1.74) | 1.77 (1.65–1.89) |
| Not performed | 0.69 (0.66–0.71) | 0.98 (0.94–1.02) |
| **Type of entry** |  |  |
| New case | Reference | Reference |
| Relapse | 1.16 (1.12–1.19) | 1.07 (1.04–1.11) |
| Reentry after abandonment | 1.49 (1.45–1.54) | 1.21 (1.17–1.24) |
| Unknown | 2.76 (2.53–3.02) | 1.94 (1.77–2.13) |
| Transfer | 1.14 (1.10–1.19) | 1.07 (1.03–1.12) |
| **Comorbid conditions** |  |  |
| HIV | 5.96 (5.84–6.08) | 5.16 (5.05–5.27) |
| Alcohol use disorder | 1.30 (1.28–1.33) | 1.44 (1.41–1.47) |
| Diabetes | 0.94 (0.91–0.96) | 1.07 (1.03–1.10) |
| Mental illness | 1.42 (1.36–1.49) | 1.23 (1.17–1.29) |
| **In prison (at diagnosis)** |  |  |
| No | Reference | Reference |
| Yes | 0.57 (0.54–0.59) | 0.67 (0.64–0.70) |
| Unknown | 1.06 (1.02–1.10) | 1.05 (1.01–1.09) |

* TB episode defined as < 12 months after TB diagnosis. CI = confidence interval. All results are adjusted for the interaction between age group and years since TB, along with race, state, and educational attainment.

**Table S8. Cumulative mortality (%) by year since diagnosis and age group**

|  |  | **Year 1** | **Year 2** | **Year 5** | **Year 10** |
| --- | --- | --- | --- | --- | --- |
| **General population** | **Overall** | 0.69 | 1.41 | 3.69 | 7.97 |
|  | **Age group** |  |  |  |  |
|  | 0-14 | 0.14 | 0.19 | 0.34 | 0.72 |
|  | 15-29 | 0.18 | 0.36 | 0.91 | 1.86 |
|  | 30-44 | 0.27 | 0.55 | 1.47 | 3.34 |
|  | 45-59 | 0.71 | 1.46 | 3.95 | 9.12 |
|  | 60-74 | 2.01 | 4.11 | 11.07 | 24.87 |
|  | 75-89 | 6.34 | 12.70 | 31.52 | 59.56 |
| **TB cohort** | **Overall** | 6.82 (6.77–6.86) | 8.75 (8.67–8.82) | 12.87 (12.73–13.01) | 17.88 (17.55–18.21) |
|  | **Age group** |  |  |  |  |
|  | 0-14 | 2.42 (2.24–2.60) | 2.89 (2.63–3.16) | 3.77 (3.31–4.29) | 4.87 (3.92–6.21) |
|  | 15-29 | 2.88 (2.82–2.95) | 3.99 (3.88–4.10) | 6.29 (6.06–6.52) | 8.94 (8.40–9.52) |
|  | 30-44 | 6.50 (6.41–6.60) | 8.64 (8.49–8.79) | 13.10 (12.79–13.41) | 18.44 (17.72–19.18) |
|  | 45-59 | 9.40 (9.27–9.53) | 12.16 (11.96–12.36) | 18.76 (18.35–19.18) | 27.76 (26.74–28.81) |
|  | 60-74 | 14.38 (14.15–14.61) | 18.33 (17.98–18.69) | 27.28 (26.57–28.00) | 39.47 (37.77–41.25) |
|  | 75-89 | 22.81 (22.30–23.31) | 27.99 (27.22–28.75) | 39.88 (38.46–41.33) | 53.99 (50.84–57.27) |
| **Excess mortality** | **Overall** | 6.12 (6.07–6.17) | 7.34 (7.27–7.42) | 9.18 (9.04–9.32) | 9.90 (9.58–10.24) |
|  | **Age group** |  |  |  |  |
|  | 0-14 | 2.28 (2.10–2.46) | 2.70 (2.45–2.98) | 3.43 (2.97–3.95) | 4.16 (3.20–5.49) |
|  | 15-29 | 2.70 (2.64–2.77) | 3.63 (3.52–3.74) | 5.38 (5.15–5.61) | 7.08 (6.54–7.65) |
|  | 30-44 | 6.23 (6.14–6.33) | 8.09 (7.94–8.24) | 11.63 (11.32–11.93) | 15.09 (14.37–15.84) |
|  | 45-59 | 8.69 (8.57–8.82) | 10.70 (10.50–10.90) | 14.81 (14.40–15.22) | 18.63 (17.62–19.68) |
|  | 60-74 | 12.37 (12.14–12.60) | 14.22 (13.86–14.58) | 16.21 (15.50–16.92) | 14.60 (12.89–16.38) |
|  | 75-89 | 15.37 (15.96–16.97) | 15.28 (14.52–16.05) | 8.36 (6.93–9.81) | -5.57 (-8.71– -2.28) |

Values in parentheses represent 95% uncertainty intervals.

**Table S9. Cumulative and excess mortality (%) by year since diagnosis and sex**

|  |  | **Year 1** | **Year 2** | **Year 5** | **Year 10** |
| --- | --- | --- | --- | --- | --- |
| **General population** | **Overall** | 0.69 | 1.41 | 3.69 | 7.97 |
|  | Female | 0.47 | 0.97 | 2.59 | 5.76 |
|  | Male | 0.80 | 1.62 | 4.22 | 9.04 |
| **TB cohort** | **Overall** | 6.82 (6.77–6.86) | 8.75 (8.67–8.82) | 12.87 (12.73–13.01) | 17.88 (17.55–18.21) |
|  | Female | 5.43 (5.35–5.50) | 6.82 (6.71–6.93) | 9.50 (9.30–9.70) | 12.71 (12.28–13.15) |
|  | Male | 7.46 (7.40–7.52) | 9.65 (9.56–9.75) | 14.47 (14.28–14.66) | 20.36 (19.93–20.79) |
| **Excess mortality** | **Overall** | 6.12 (6.07–6.17) | 7.34 (7.27–7.42) | 9.18 (9.04–9.32) | 9.90 (9.58–10.24) |
|  | Female | 4.95 (4.88–5.03) | 5.85 (5.74–5.96) | 6.91 (6.72–7.11) | 6.95 (6.52–7.40) |
|  | Male | 6.66 (6.60–6.72) | 8.03 (7.94–8.13) | 10.25 (10.06–10.43) | 11.32 (10.90–11.76) |

Values in parentheses represent 95% uncertainty intervals.

**Table S10. Cumulative mortality (%) by year since diagnosis and region**

|  |  | **Year 1** | **Year 2** | **Year 5** | **Year 10** |
| --- | --- | --- | --- | --- | --- |
| **General population** | **Overall** | 0.69 | 1.41 | 3.69 | 7.97 |
|  | **Region** |  |  |  |  |
|  | North | 0.67 | 1.36 | 3.57 | 7.72 |
|  | Northeast | 0.74 | 1.51 | 3.93 | 8.47 |
|  | Central-West | 0.80 | 1.62 | 4.21 | 9.04 |
|  | Southeast | 0.67 | 1.35 | 3.55 | 7.69 |
|  | South | 0.67 | 1.36 | 3.57 | 7.78 |
| **TB cohort** | **Overall** | 6.82 (6.77–6.86) | 8.75 (8.67–8.82) | 12.87 (12.73–13.01) | 17.88 (17.55–18.21) |
|  | **Region** |  |  |  |  |
|  | North | 5.79 (5.64–5.93) | 7.39 (7.17–7.60) | 10.58 (10.17–11.00) | 14.34 (13.42–15.33) |
|  | Northeast | 6.43 (6.34–6.52) | 8.33 (8.19–8.47) | 12.36 (12.10–12.63) | 17.09 (16.49–17.71) |
|  | Central-West | 7.41 (7.17–7.64) | 9.50 (9.14–9.86) | 13.80 (13.13–14.48) | 18.56 (17.10–20.16) |
|  | Southeast | 6.70 (6.63–6.78) | 8.57 (8.46–8.69) | 12.74 (12.53–12.96) | 18.08 (17.59–18.59) |
|  | South | 8.62 (8.47–8.77) | 11.07 (10.85–11.30) | 15.96 (15.53–16.39) | 21.61 (20.67–22.60) |
| **Excess mortality** | **Overall** | 6.12 (6.07–6.17) | 7.34 (7.27–7.42) | 9.18 (9.04–9.32) | 9.90 (9.58–10.24) |
|  | **Region** |  |  |  |  |
|  | North | 5.11 (4.97–5.26) | 6.02 (5.81–6.24) | 7.00 (6.60–7.42) | 6.62 (5.69–7.61) |
|  | Northeast | 5.69 (5.60–5.78) | 6.83 (6.69–6.97) | 8.43 (8.16–8.70) | 8.62 (8.02–9.24) |
|  | Central-West | 6.61 (6.38–6.85) | 7.88 (7.53–8.24) | 9.59 (8.92–10.27) | 9.52 (8.06–11.12) |
|  | Southeast | 6.04 (5.97–6.11) | 7.22 (7.11–7.33) | 9.19 (8.98–9.40) | 10.39 (9.90–10.90) |
|  | South | 7.95 (7.80–8.10) | 9.71 (9.49–9.94) | 12.38 (11.96–12.81) | 13.84 (12.89–14.83) |

Values in parentheses represent 95% uncertainty intervals.

**Table S11.** Detailed causes of CVD-related deaths among TB survivors (n = 11,555)

| **Cause** | **Frequency** | **Percentage (%)** |
| --- | --- | --- |
| Acute myocardial infarction | 2,735 | 23.7 |
| Cerebrovascular diseases | 2,716 | 23.5 |
| Hypertension | 1,188 | 10.3 |
| Heart failure | 1,153 | 10.0 |
| Cardiomyopathy | 791 | 6.9 |
| Chronic ischemic heart disease | 537 | 4.7 |
| Pulmonary embolism | 359 | 3.1 |
| Complications and ill-defined descriptions of heart disease | 297 | 2.6 |
| Hypertensive chronic kidney disease | 232 | 2.0 |
| Other | 1,547 | 13.4 |

**Table S12.** Detailed causes of respiratory-related deaths (excl. TB) among TB survivors (n = 13,493)

| **Cause** | **Frequency** | **Percentage (%)** |
| --- | --- | --- |
| Pneumonia | 5,465 | 40.5 |
| Chronic Obstructive Pulmonary Disease | 3,755 | 27.8 |
| Other interstitial pulmonary diseases | 544 | 4.0 |
| Emphysema | 491 | 3.6 |
| Respiratory failure | 386 | 2.9 |
| Pulmonary edema | 213 | 1.6 |
| Bronchiectasis | 188 | 1.4 |
| Asthma | 156 | 1.2 |
| Pneumoconiosis due to dust containing silica | 154 | 1.1 |
| Pneumonitis due to inhalation of food and vomit | 151 | 1.1 |
| Pyothorax | 122 | 0.9 |
| Pleural effusion | 112 | 0.8 |
| Other respiratory disorders | 1,756 | 13.0 |

**Figure S1. Mortality rate ratios for individuals with TB by year since diagnosis and sex, as compared to the general population**

**
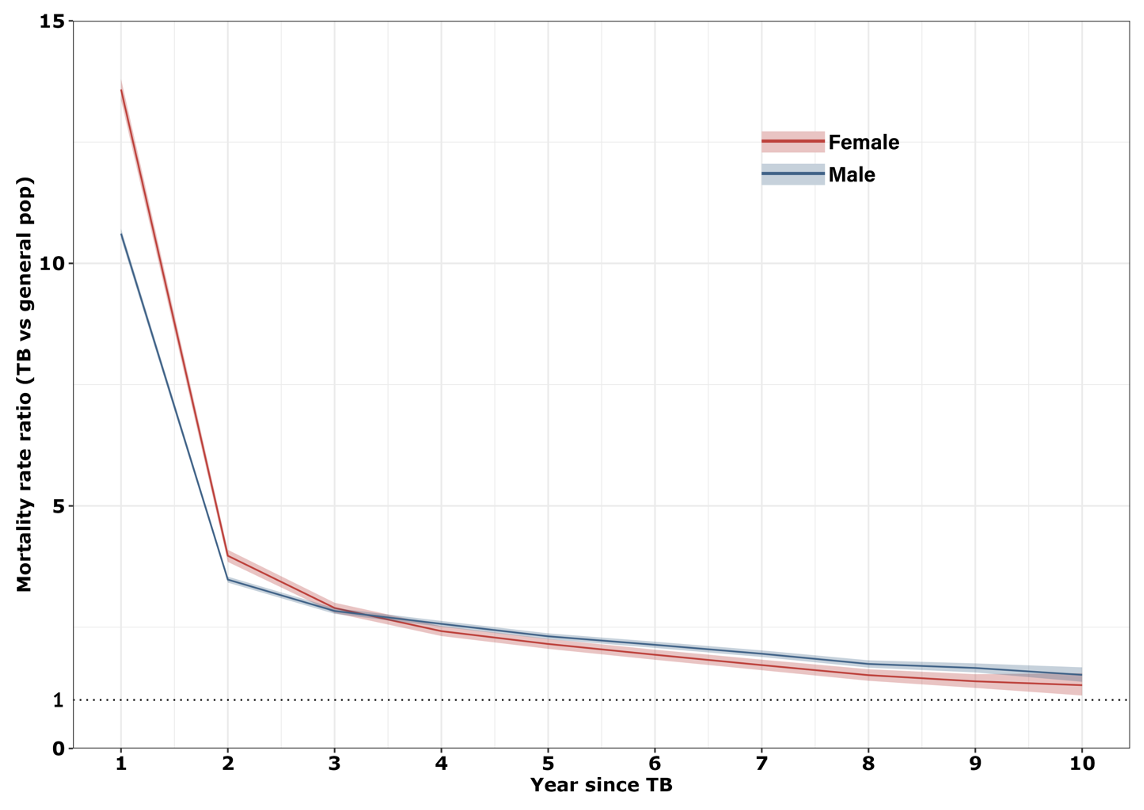
**

Lines represent point estimates, shaded regions represent 95% uncertainty intervals.

**Figure S2. Mortality rate ratios for individuals with TB by year since diagnosis and by region, as compared to the general population.**

**
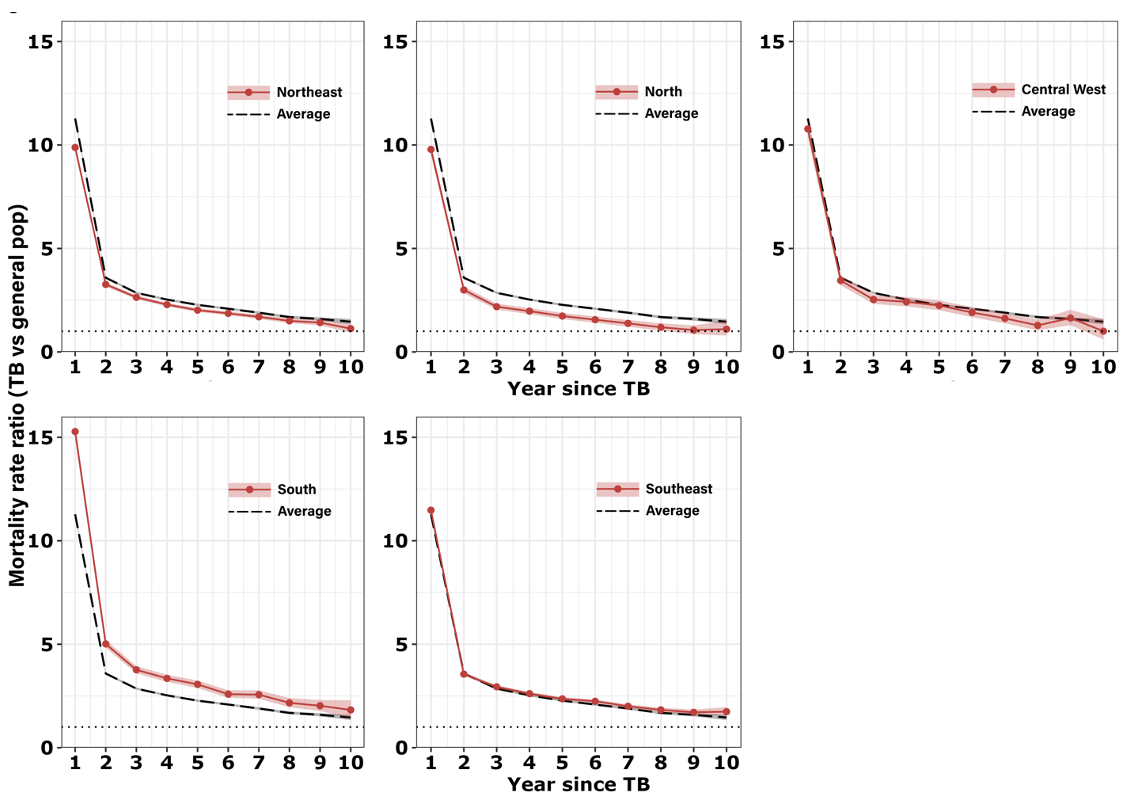
**

Lines represent point estimates, shaded regions represent 95% uncertainty intervals.

**Figure S3. Cumulative mortality by age group**

**
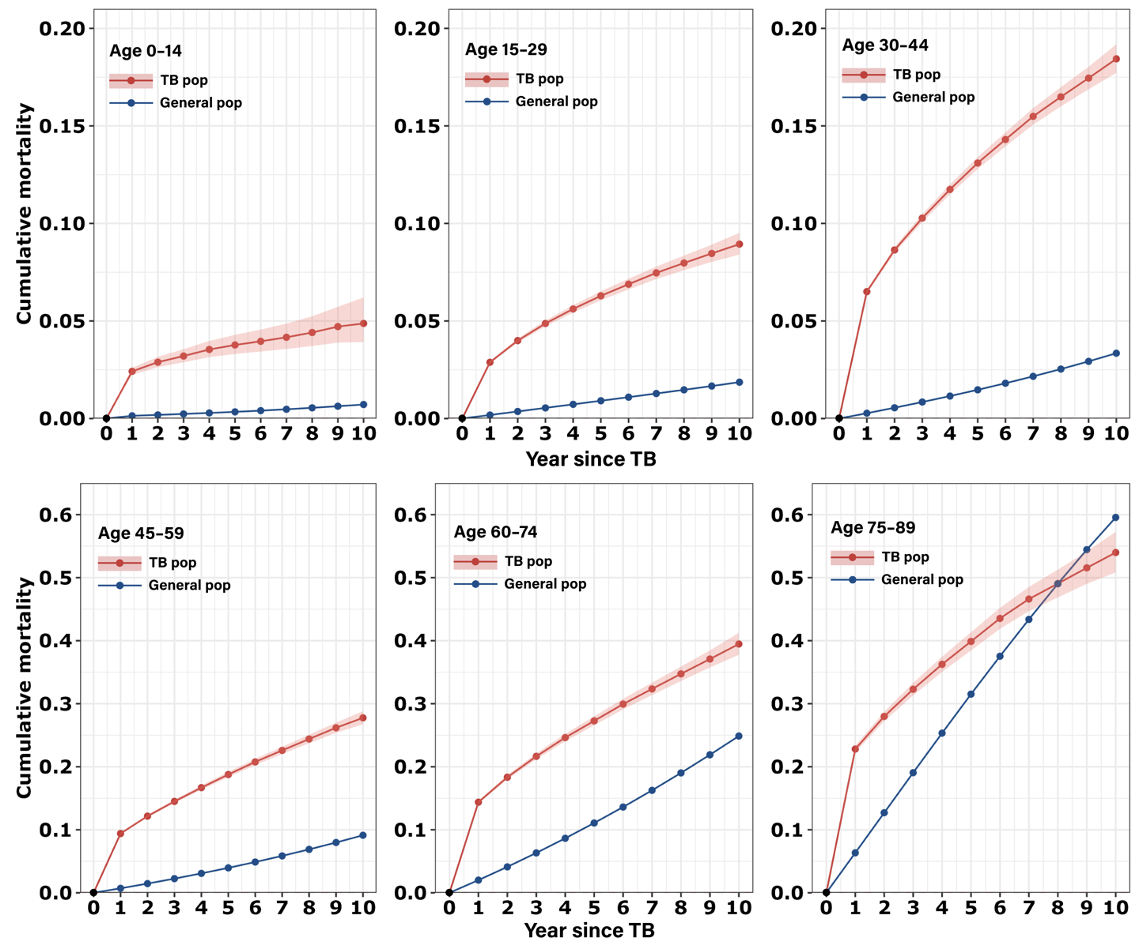
**

Lines represent point estimates, shaded regions represent 95% uncertainty intervals.

**Figure S4. Cumulative mortality by sex**

**
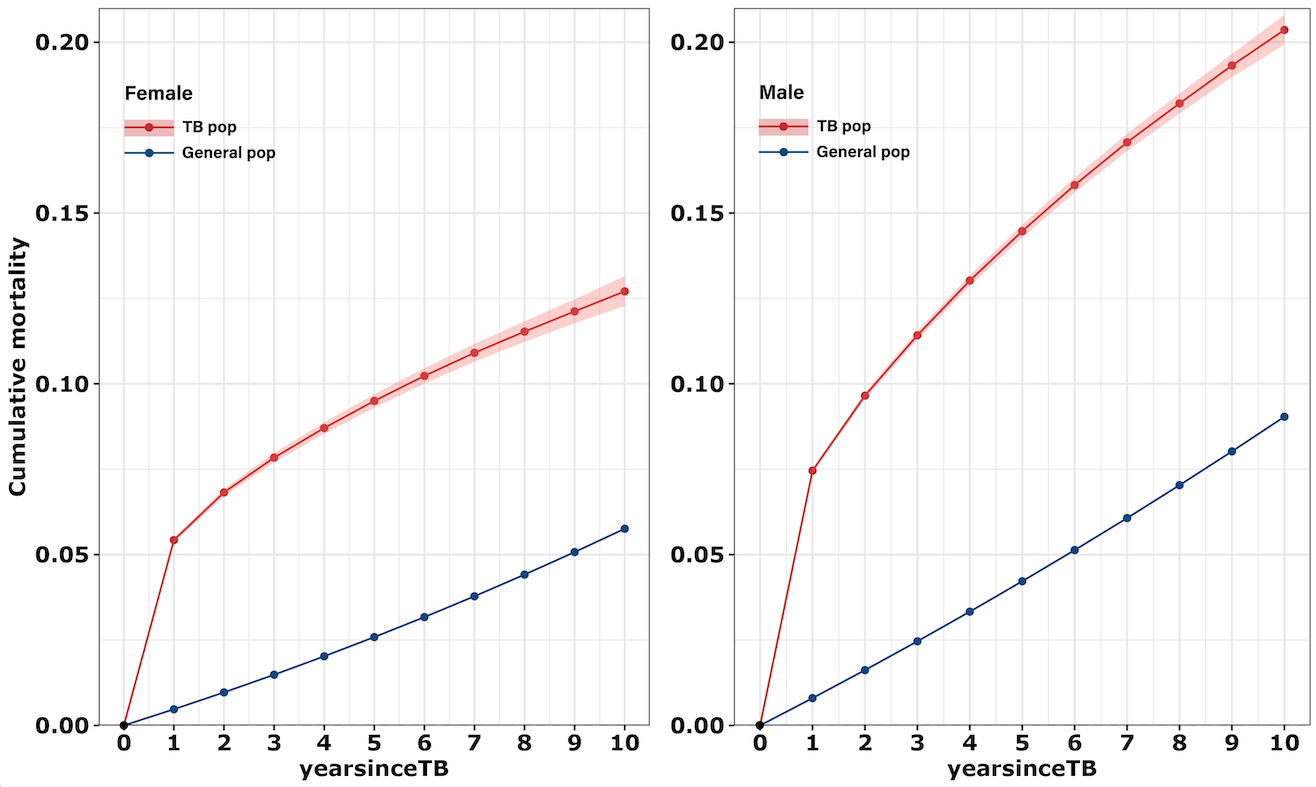
**

Lines represent point estimates, shaded regions represent 95% uncertainty intervals.

**Figure S5. Cumulative mortality by region**


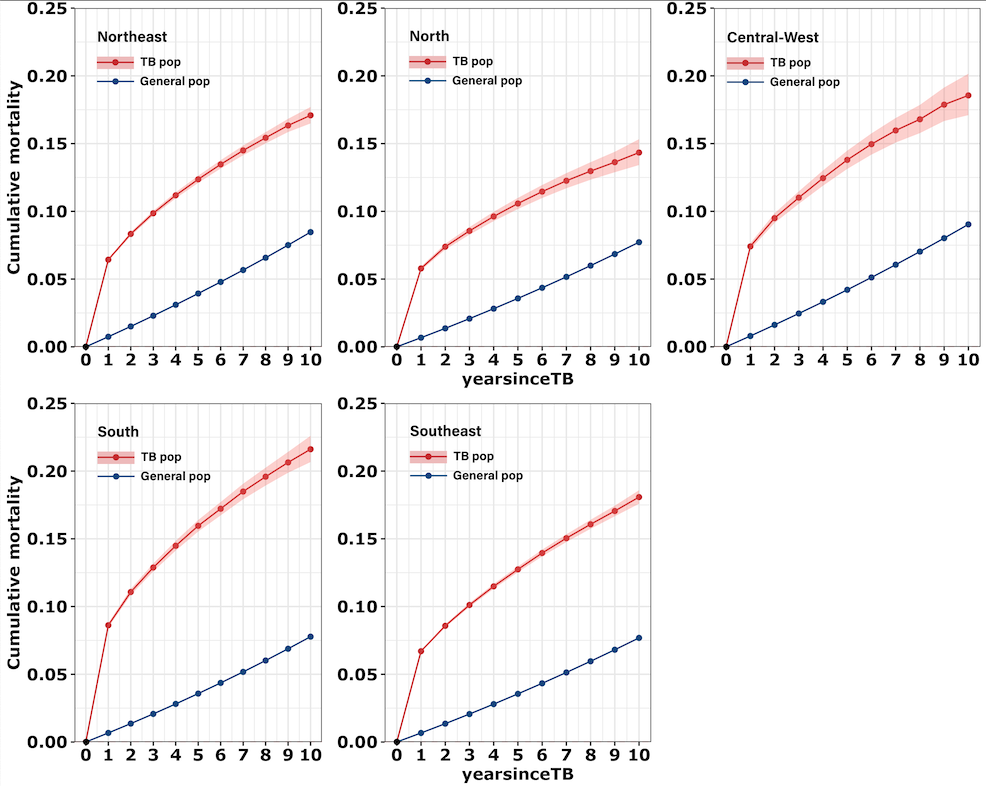


Lines represent point estimates, shaded regions represent 95% uncertainty intervals.

**Figure S6. Distribution of cause of death in the TB cohort compared to a matched general population sample, by years since TB diagnosis, by region.**

**
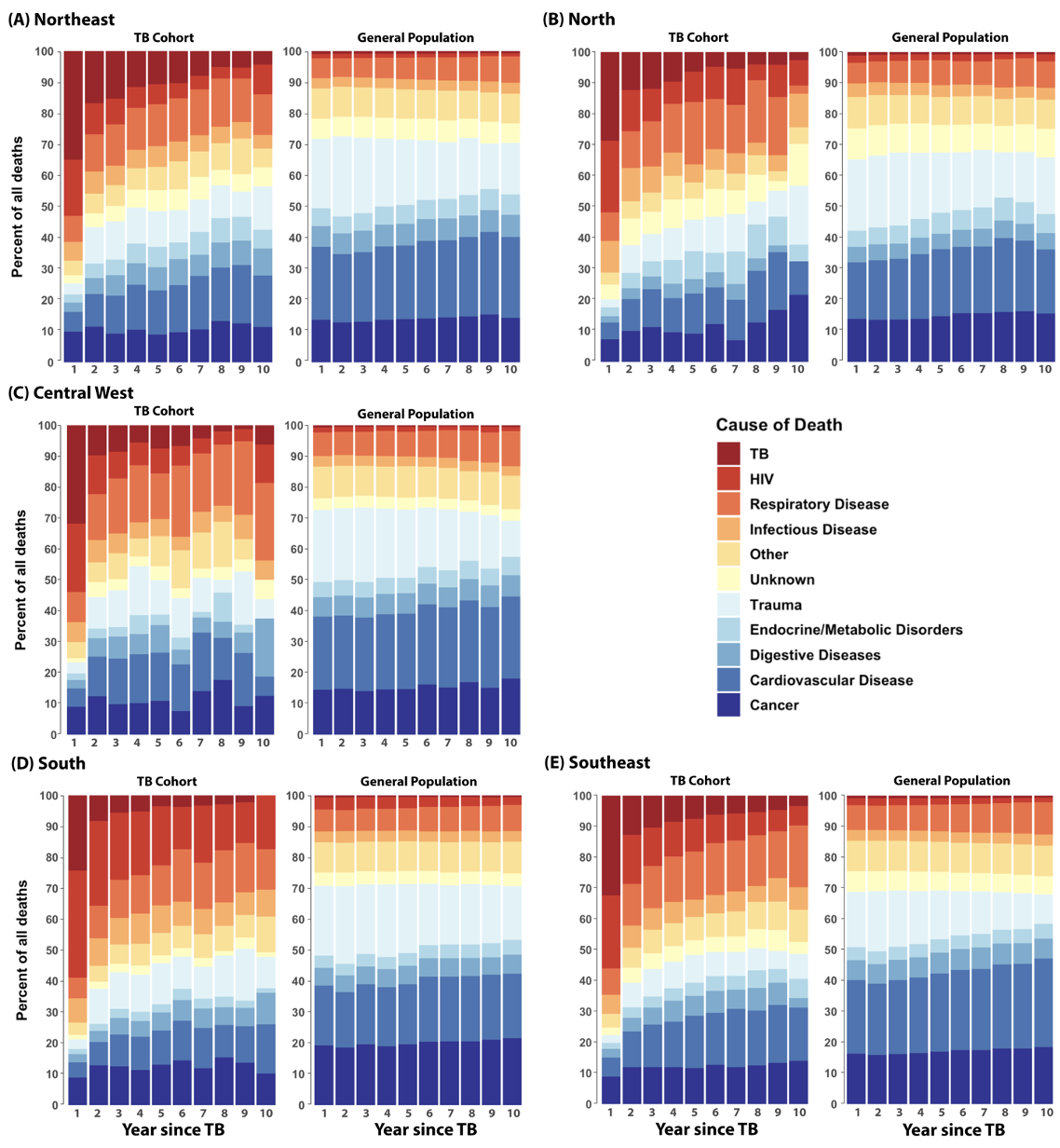
**
